# Towards sensor array materials: can failure be delayed?

**DOI:** 10.1088/1468-6996/16/3/034607

**Published:** 2015-06-02

**Authors:** Samir Mekid, Nouari Saheb, Shafique M A Khan, Khurram K Qureshi

**Affiliations:** 1 Mechanical Engineering Department, King Fahd University of Petroleum and Minerals, Dhahran, Saudi Arabia; 2 CENT, King Fahd University of Petroleum and Minerals, Dhahran, Saudi Arabia; 3 Electrical Engineering Department, King Fahd University of Petroleum and Minerals, Dhahran, Saudi Arabia

**Keywords:** smart materials, nervous materials, embedded sensors, sensor materials

## Abstract

Further to prior development in enhancing structural health using smart materials, an innovative class of materials characterized by the ability to feel senses like humans, i.e. ‘nervous materials’, is discussed. Designed at all scales, these materials will enhance personnel and public safety, and secure greater reliability of products. Materials may fail suddenly, but any system wishes that failure is known in good time and delayed until safe conditions are reached. Nervous materials are expected to be the solution to this statement. This new class of materials is based on the novel concept of materials capable of feeling multiple structural and external stimuli, e.g. stress, force, pressure and temperature, while feeding information back to a controller for appropriate real-time action. The strain–stress state is developed in real time with the identified and characterized source of stimulus, with optimized time response to retrieve initial specified conditions, e.g. shape and strength. Sensors are volumetrically embedded and distributed, emulating the human nervous system. Immediate applications are in aircraft, cars, nuclear energy and robotics. Such materials will reduce maintenance costs, detect initial failures and delay them with self-healing. This article reviews the common aspects and challenges surrounding this new class of materials with types of sensors to be embedded seamlessly or inherently, including appropriate embedding manufacturing techniques with modeling and simulation methods.

## 1. Introduction

**Figure 1.**
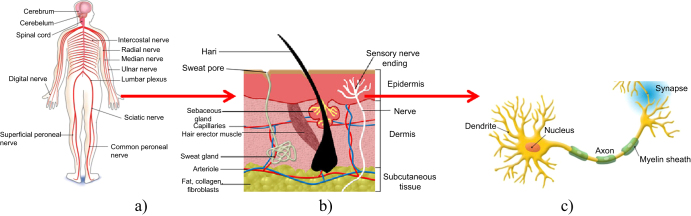
The biological neural system: (a) the nervous system of a human, (b) the visual cortex, (c) a simplified illustration of the biological nervous system.

The introduction to nervous materials requires an understanding of the complexities of the human sensory and nervous system. The latter is a living organism that senses the environment and responds appropriately. This system is exceedingly complex, with a large number of components and an extremely large number of interactions between these components. The system is inherently seamless. The human body contains an incredible sensory system, information transfer mechanism, and sensory information processing unit (brain), shown in figure 1. The sensations in a human body occur when external stimuli interact with sensory receptors. Most of the sensory receptors are distributed in the skin and tissues of the body. Hence, the skin can feel pain, temperature, pressure, and other stimuli via these nerve cells in tissues. The sensory information is transferred by means of electrical signals because the neuron is sufficiently composed of multiple analogue inputs (called *dendrites*) and a digital output (called an *axon*) like an electrical device (figure 1). The sensory information converges on spinal nerves then enters the brain as trains of action potentials traveling along individual sensory neurons to evaluate the stimuli. Sundaresan and Shultz [1] commented on the purpose of structural health monitoring: ‘for the purpose of structural health monitoring, we can consider the following properties of a neural system:

(1) information proceeds through the system through different levels;
(2) information is processed at each level; the level of processing is higher the farther away from the input;
(3) information is synthesized at each level, and a decision is made as to what is sent forward to the next level;
(4) the system has mechanisms for noise suppression. There is also interconnection between sensory cortices’.

Hence, nervous materials will have a surface embedded network of sensors that will not affect the integrity of the material but monitor its behavior and possibly react to correct or delay any possible failure. This will be classified as a new class of materials with various possible names, e.g. nervous or sensorial materials. In essence, such a material can ‘feel’ its environment and react to correct or delay failure using embedded actuators.

With an ever-changing environment, the world is becoming complex, requiring more stringent sets of smart design to ensure personnel and public safety through products at all scales, e.g. the high integrity of aircraft, civil infrastructures, nuclear energy and high-tech aerospace/aircraft structures are just a few examples. Two major sets of design requirements are targeted: damage tolerance to preclude structural safety problems and durability to preclude expensive maintenance and repairs. This also means a need for a reactive system to any environmental change, accidental effect or expected failure. Early detection and correction of the structural damage or deterioration prior to local failure can prevent catastrophic failures; hence, a new class of materials is required with specific features and characteristics. This new generation of materials is required to have a nervous character and be part of a smart design, avoiding standard material configuration as well as dense deployment of sensors and actuators with narrow coverage and difficult-to-access locations. Active research and systems are currently being undertaken to deliver some output [2–4].

We expect to build a human-like nervous system in the core material used for frames and structures such as aircraft, cars and other sensitive applications able to capture the stress or ‘pain’ and possible failure of multi-parameters and immediately feedback to an intelligent controller for instant decision making. The nervous material, as a unit panel or block design, will have the ability to monitor large regions of the structure by connecting to each other with sensor clusters. Due to this advantage, it will be potentially possible to:

(1) reduce maintenance cost,
(2) perform an early failure detection in real time,
(3) monitor a product that has a large and/or sensitive structure e.g. aircraft or nuclear power plant, and
(4) compensate for any deformation and prevent failure (self-healing).

Self-healing as a feature for short or long periods has become a hot research topic due to its importance.

**Figure 2.**
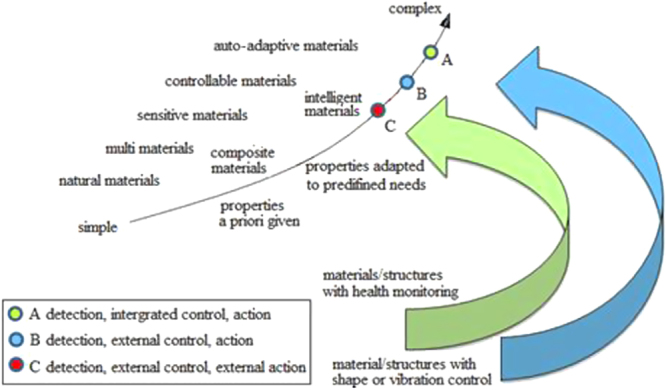
General evolution of materials/structures used by people, and the place of smart structures. Reprinted from [3], copyright 1989, with permission from Elsevier.

The concept of smart/intelligent materials and structures can be considered as a step in the general evolution of man-made objects as shown in figure 2 [3]. This schematic shows a continuous trend from simple materials to complex materials in man-made production. A simple material, which is a homogeneous material, made of natural properties, becomes complex by developing the technology of multi-materials (in particular, composite materials) that allow for the design of new structures with functional properties adapted to specific applications.

Recently, significant progress has been achieved in the area of structural health monitoring [5]. Materials with health monitoring systems exhibit several advantages including monitoring all aspects related to damages, loads, conditions, etc. However, these materials have limitations such as robust, effective, self-healing and reliable monitoring characteristics. Vision using structural health monitoring has two aspects: an intelligent sensing network through the structure being monitored, or embedded intelligence in the materials composing the structure. New structures that are made for specific needs should adapt to changing environmental conditions. This requires making them sensitive, controllable and active. Therefore, materials/structures are getting ‘smarter’ with a variety of functional properties. In other words, such materials could also be composed of multi-functional materials that are integrated with sensors, actuators, electronics and communication systems to decide actions at any environmental change. The structural conditions and residual life of a structure will be monitored and predicted by sensors and intelligent diagnostics. Moreover, an embedded sensory network and nervous system would enable structures to have intelligent communication. Due to the intelligent communication system, the structural conditions will be monitored in real time. Because of this advantage, the structure will be more adaptive to the changing environment. Hence, damage will be mitigated and catastrophe prevented with embedded sensors, actuators and multi-functional materials. For example, unmanned aerial vehicles (UAVs) must function reliably after long-term storage, requiring a nervous system integral to the airplane, which will add both complexity and cost. Sensors developed for one purpose can often be adapted to serve other sensory functions. In some cases, they can also serve as actuators. Sensor types and their embedding possibility, as well as embedding techniques and their related challenges, are discussed next with further concluding remarks.

## 2. System descriptions

### 2.1. Sensor system

As an initial approach, sensors were considered to be selected from those already in existence e.g. microelectromechanical systems (MEMS), fiber optics and fiber Bragg gratings (FBGs), except for nanoelectromechanical systems (NEMS) where it is believed that research can focus on development for this purpose because of the size aspect. For any underperformance, a new type of sensor should be developed to achieve a human-like high performance feeling and reaction. Biological nervous systems can perceive, comprehend and respond to environmental stimuli very precisely. It is known that a large number of homogeneous sensors with diverse sensing capabilities have been embedded in biological systems. Therefore, multiple stimuli (temperature, pressure, humidity etc) can be sensed simultaneously and precisely. However, we are far from satisfying this condition for nervous materials because of the limited performance of the existing sensors. The efficiency of nervous materials can be improved by embedding the appropriate sensors [6, 7]. In fact, realization of nervous materials depends on the development and integration of sensors in the host materials with large-scale coverage in a network [8]. The most important features of the sensors suitable for nervous materials are size, weight and sensing resolution. Small size and weight of sensors will introduce fewer wounds in the host materials during integration [9]. The sensor networks can be either wired or wireless electronic sensor networks, which have the following restrictions.

• Wired sensor networks are susceptible to electromagnetic interference. Moreover, electric discharge and lightening can affect the conductive path and electronic devices, which require costly and bulky shielding, a disadvantage for certain structures such as aircraft.
• A local energy supply is needed for wireless sensor networks, which cannot be replaced after being embedded into the materials. This energy dependency can affect the long-term performance of nervous materials, while energy harvesting can be used depending on the application in terms of power levels and available harvesting techniques.

On the other hand, fiber optic sensors have some distinguishing advantages over conventional electronic sensors [10–12].

(1) Fiber optic geometry can be easily embedded into the nervous materials introducing fewer wounds in the host materials.
(2) Fiber optic sensors can tolerate high temperature and pressure during the manufacturing of the nervous system.
(3) The dielectric nature of the optical fiber provides a high degree of immunity to electromagnetic interference. This also helps to protect it from lightening and electric discharge hazards.
(4) Many fiber optic sensors can be multiplexed in a single line.
(5) Fiber optic sensors are compatible with a fiber optic data link, which can provide the necessary bandwidth for a large number of sensors.

In general, fiber optic sensors can be divided into three types: interferometric, distributed and grating-based sensors [13].

#### 2.1.1. Interferometric sensor

In an interferometric sensor, a physical change in structure is reflected by the phase change of the two interfering light signals [14]. A Fabry–Perot interferometric sensor can achieve a maximum strain resolution of 0.15 *μ∊*. The typical measurement range is ±1000 *μ∊*, which can be extended up to ±5000 *μ∊*. It can be operated at temperatures ranging from −40 °C to +250 °C. Moreover, Fabry–Perot interferometric sensors are very compact with a length from 1–20 mm and can be embedded into the materials without any weight penalty or adverse effects. However, the multiplexing capability of this sensor is very poor.

#### 2.1.2. Distributed fiber optic sensor

Distributed fiber optic sensors can be divided into three categories: optical time-domain reflectometry (OTDR), Raman optical time-domain reflectometry (ROTDR) and Brillouin optical time-domain reflectometry (BOTDR). OTDR uses Rayleigh scattering to reflect the attenuation profiles along the fiber link [15]. A light pulse is launched into the optical fiber and Rayleigh backscattered light is measured to determine fiber loss, break locations, splice locations and connector locations.

Distributed sensing using ROTDR and BOTDR uses the nonlinear properties of the optical fiber. In these sensing mechanisms additional spectral components are produced, which are affected by the external environmental stimuli. The external environmental stimuli are measured by evaluating the spectral content appropriately. The Raman-scattering phenomenon is used in ROTDR, where both Stokes and anti-Stokes components are generated [16]. Temperature information of any point along the fiber link can be obtained by using the ratio of the anti-Stokes to Stokes components. ROTDR can only measure temperature with a resolution of 0.2 °C. In this technique, the maximum sensing distance is 8 km with a spatial resolution of 1 meter.

In BOTDR, a light pulse incident on the optical fiber link is partially reflected back due to Brillouin-scattering phenomena [17]. This scattering of light depends on temperature and strain applied on the fiber. In this technique, measurement distance is usually up to 30 km. The spatial resolution is from 1 to 4 meters.

#### 2.1.3. Grating-based sensor

**Figure 3.**
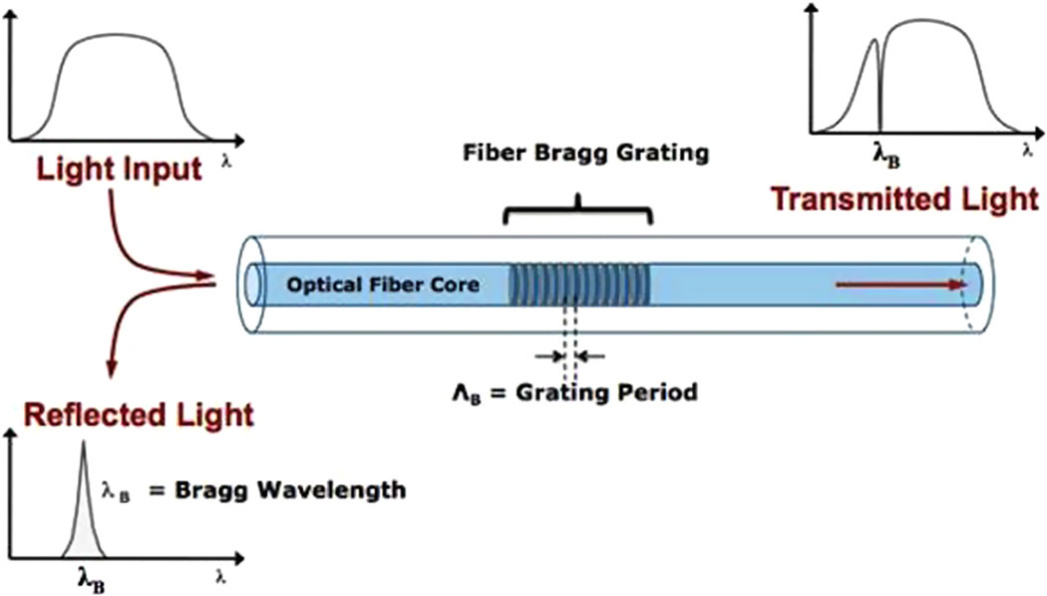
Optical fiber core with FBG.

In grating-based sensors, FBG sensors are widely used in different applications because of their outstanding characteristics and properties such as light weight, immunity to electromagnetic interference, stability and little signal loss over very long distances [18]. In this technique, an incoming light signal is reflected back at a particular wavelength, which is known as the Bragg wavelength, depending on the grating pitch and refractive index of the fiber (figure 3). Temperature, strain, vibration and other parameters can change the grating pitch. Therefore, by monitoring the shifted Bragg wavelength, several measurands can be measured. The advantages of the FBG sensors are low cost, compact size and good linearity. Moreover, the multiplexing capability of multiple wavelengths in a single fiber has made it more attractive.

They can be easily embedded into composite materials [19, 20] because of their flexibility, strength and heat resistance. Table 1 [21] provides a summary of the mechanical and physical properties of different types of optical fibers which can be used as sensors. Depending on the specific application and operating conditions, suitable optical fibers can be selected. A list of measurands that can be monitored using optical fiber sensors is presented in table 2 [21]. FBGs have the most widespread use in composites [22–27] as well as in metals [28] and alloys [29]. They attracted the serious attention of researchers because of their great potential to be used in structural health monitoring (SHM).

**Table 1.** Properties of some optical fiber materials [21]. PMMA stands for poly(methyl methacrylate).

Property	Chalcogenide	Fluoride	Sapphire	AgBr/Cl	Silica	PMMA
Spectral range	3–10 *μ*m	0.5–4.3 *μ*m	0.2–4 *μ*m	3.3–15 *μ*m	0.2–4 *μ*m	0.4–0.8 *μ*m
	3300–1000 cm^−1^	20 000–2325 cm^−1^	50 000–2500 cm^−1^	3000–667 cm^−1^	50 000–2500 cm^−1^	
Attenuation	0.5 dB m^−1^ @ 6 *μ*m	0.02 dB m^−1^ @ 2.6 *μ*m	20 dB m^−1^ @ 3 *μ*m	0.7 dB m^−1^ @ 10.6 *μ*m	0.5 dB m^−1^ @ 1.5 *μ*m	30 dB m^−1^ @ 800 nm
						0.1 dB m^−1^ @ 600 nm
Refractive index	2.9	1.51	1.7	2.0	1.458	1.492
Max. use temperature (°C)	300	250	>1500	400	800	80
Approx. price (£ m^−1^)	15	—	100	40	1	1.5
Density (kg m^−3^)	4400	4610	3970	6400	2200	1190
Young’s modulus (GPa)	21	56	414	31.97	73	3.3
Thermal expansion coefficient (×10^−6^ K^−1^)	14	18.7	8.8	35	0.54	260

**Table 2.** A summary of selected measurands that can be monitored using optical fiber sensors [21].

Measurand	Optical fiber-based sensor technique
Strain	(i) Raman
	(ii) Interferometry
	—Michelson
	—Fabry–Perot
	(iii) Intensity
	(iv) Polarization
	(v) Bragg gratings
Temperature	(i) Fluorescence
	(ii) Bragg gratings
	(iii) Interferometry
	(iv) Brillouin
Flow	Doppler
Acoustic emission	Fused tapered coupler
Pressure	Interferometry
Mixing	Scattering
Chemical concentration	(i) IR spectroscopy
	—mid-infrared
	—near infrared
	—evanescent wave
	(ii) Raman spectroscopy
Damage detection: fiber fracture	Reinforcing fiber light guides

Traditional SHM systems use wired sensors, which are time consuming to install and are relatively expensive [30–32]. Moreover, a dense array of sensors is required for reliable monitoring of local and global damage of a large structure. In the conventional approach, a large number of connecting wires may be the major obstacle in terms of scalability. Therefore, few SHM systems use wired connection for connecting a large number of sensors. On the other hand, rapid development of sensors, wireless communication, MEMS and information technology can have a significant impact on SHM. In wireless monitoring, low cost wireless sensors allow dense installation of sensor networks, which permit global to component level monitoring. In this process, a large amount of data is generated where a portion of data processing is done at the embedded microprocessor on a local sensor level. This reduces the amount of information to be transmitted over the network wirelessly. Limited transmission bandwidth and portable power supply are the major drawbacks of wireless SHM. Considering power consumption as a major limitation of wireless sensors, power-free wireless sensors known as radio frequency identification (RFID) sensors have been developed [33]. In this technology, the RFID sensor collects radio energy from a remote reader, and then acts as a passive transponder to emit microwave or ultra-high frequency radio waves. The distinguishing advantage of this technique is the indefinite lifetime of wireless sensors. However, RFID-based technologies offer a limited range of applications (distance between reader and device is <5 m). This range is significantly reduced when the sensors are embedded in the structures.

#### 2.1.4. MEMS

MEMS are made by integrating mechanical elements, sensors, actuators and electronics on a common silicon substrate [34]. Classical sensors and actuators provide a very limited range of applications in high temperature and pressure conditions. The development of smart products/materials is possible by increasing the computational abilities of microelectronics with active perception and adaptive control abilities. Precise monitoring can be achieved by using small and versatile new generation MEMS sensors. Moreover, MEMS with new generation actuators will provide innovative controlling methods. Altogether, MEMS technology can provide one missing component of the smart products/materials, which have the ability to comprehend the surrounding environment and operating conditions, and consequently adjust them either to obtain optimal performance, or to increase their reliability by identifying and avoiding failures, by means of both new generation sensors and actuators.

Recently, considerable attention has been given to the use of polymers in microelectronics and MEMS. Moldability, conformability, ease in deposition in the form of thin and thick films, semiconducting and even metallic behavior in selected polymers, a choice of widely different molecular structures and the possibility of piezoelectric and pyroelectric effects in the polymer side-chain have made them more attractive in MEMS systems [35]. Some polymer materials need to have conductive, piezoelectric or ferroelectric effects for some MEMS devices. For polymeric MEMS, polymers should have the following properties:

• good interfacial adhesion between the various polymer layers;
• appropriate elastic moduli to support the deformation required in MEMS;
• excellent overall dimensional stability;
• long-term environmental stability.

#### 2.1.5. Carbon nanotubes

Carbon nanotubes (CNTs) have some exceptional properties (mechanical, electrochemical, piezoresistive), which make them suitable for smart nanoscale materials [36, 37]. Recently, CNTs have aroused much interest among researchers wishing to develop advanced sensors and actuators for structural applications. The size, mechanical strength and electrical properties of the nanotubes are strongly determined by the structure of the materials. For example, the ductility and electrical conductivity of armchair nanotubes are greater than zigzag nanotubes. One layer of CNTs can achieve 50 times the tensile strength of traditional steel, while the mass density of CNTs is only 1/6 that of steel. Their thermal conductivity is two times higher than diamond and electrical conductivity is 100 times higher than copper. Moreover, they are super-elastic and can be bent to large angles without breaking. In addition, variation of conductance in a nanotube depends on mechanical deformation or chemical functionalization of the surface. Extremely small sensors can be made using CNTs due to their small size. All the remarkable features of CNTs represent the potentiality of developing actuators with high stress, high strain and low operating voltage. Unlike other smart materials, CNTs can be used simultaneously as structural, functional and smart materials because of their exceptional properties.

#### 2.1.6. Piezoelectric wafer active sensors

Piezoelectric wafer active sensors (PWAS) have been considered as an important tool for SHM [38]. These sensors can send and receive guided Lamb/Rayleigh waves that scan the structure and detect the presence of emerging cracks and structural damage. These inexpensive and unobtrusive active sensors can be used in many important applications. However, existing fabrication and installation methods of these sensors on metallic structures are rather primitive. In the existing methods, pre-manufactured piezoelectric wafers are adhesively bonded to the structural surface. These bonding layers are susceptible to environmental ingression, which could induce loss of contact with the structural substrate. An acoustic impedance mismatch may also be formed between the layers, which is detrimental to structural damage detection. These shortcomings can be overcome by producing active sensors directly on the structural substrate using ferroelectric thin film and nanofabrication technology. The piezoelectric properties of ferroelectric thin films are close to those of single-crystal ferroelectrics. The piezoelectric properties of single-crystal ferroelectrics are better than traditional piezoceramics. In addition, much smaller polling voltage/power is required for the thin films. Moreover, integration of ferroelectric thin films in micro/nano electromechanical systems, tunable wireless communication elements, and other modern devices has been performed successfully. However, key challenges exist in extending this technology to SHM since the fabrication of ferroelectric thin films on structural materials (steel, aluminum, titanium, etc) has not yet been attempted.

**Figure 4.**
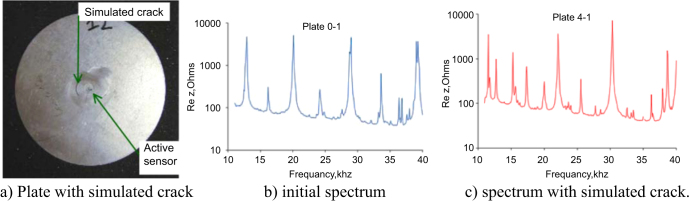
Spectral changes with one damage in the 10–40 kHz range due to a crack. Reprinted from [39].

In a plate with a simulated crack next to the PWAS sensor (figure 4(a)) a spectral measurement shows few added frequencies in figure 4(c) compared to figure 4(b).

### 2.2. Host materials

FBG sensors will be embedded in different materials that will play the role of host materials with various embedding conditions discussed hereafter.

#### 2.2.1. FBG sensors in composite structures

**Figure 5.**
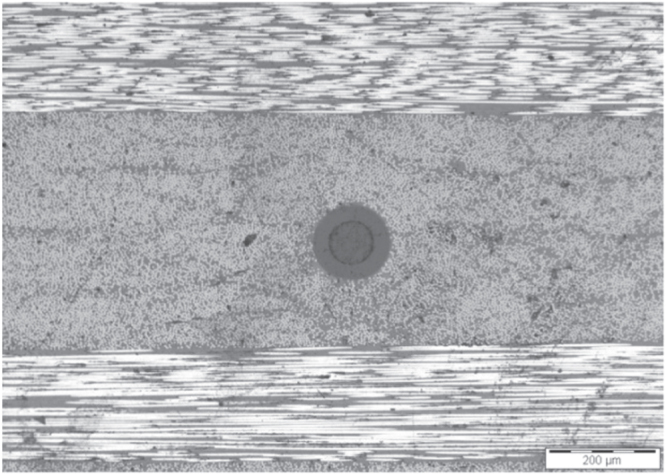
Magnified cross section of a laminated carbon fiber panel containing an embedded optical fiber sensor. Reproduced from [41], under a Creative Commons Attribution 3.0 Unported (CC BY 3.0) License, copyright 2011 MDPI AG.

Embedding FBG sensors into a polymer matrix is a relatively easy process [19, 23, 24]. The great potential of optical fiber sensors for structural health monitoring was highlighted by Minakuchi and Takeda [19]. The authors reviewed research work mainly in Brillouin-based distributed sensing, and presented damage detection, life cycle monitoring and shape reconstruction systems applicable to large-scale composite structures. In a review on strain measurements of composite laminates with embedded FBGs, Luyckx and co-authors [23] provided an overview of some of the technical issues related to embedding sensors in composite structures. The output of an embedded FBG sensor was related to the strain of the structure through a monitoring scheme. In addition, the authors focused on temperature compensation methods when measuring strain with FBGs. In addition, embedding a combination of shape memory alloy wires and FBG sensors in glass fiber reinforced composites was investigated by Ho *et al* [40]. More recently, the possibility of embedding polymer optical fiber gratings [27] in composite materials was explored, and promising results were reported at temperatures less than 70 °C [25, 26]. A simple, low cost relative humidity sensor system based on polymer fiber Bragg was developed. The sensor might have potential applications for fast and accurate real time humidity control [27]. In another work [25], researchers investigated the characteristics of polymer fiber Bragg gratings embedded in composite materials and compared them with the characteristics of their silica counterparts. The fabricated composites with embedded sensors were subjected to temperature and strain changes. The authors concluded that from the observed wavelength shift and spectral bandwidth change of the polymer FBG, temperature and thermal expansion effects in the composite material could be measured simultaneously. Figure 5 shows a magnified cross section of a laminated carbon fiber panel containing an embedded optical fiber sensor with a diameter of 0.1 mm.

#### 2.2.2. FBG sensors in metals and alloys

**Figure 6.**
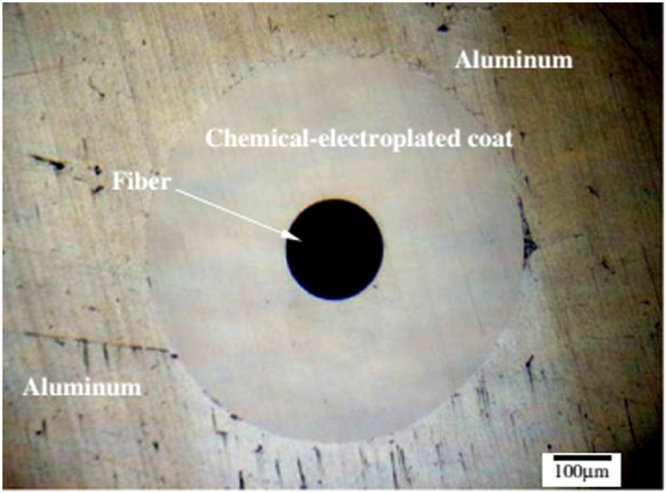
Cross section of an aluminum/chemical-electroplated fiber/aluminum sample. Reprinted from [28], copyright 2012, with permission from Elsevier.

Metallic materials remain widely used in many industries such as automotive [42] and aerospace [43]. Although FBGs were successfully embedded in composites and used in different applications, their use in metallic structures is very limited. Embedding FBG sensors in metals and alloys is more challenging because of grating degradation and sensitivity loss at high temperature [44]. Li *et al* [28] explored the possibility of embedding FBGs in Al and Cu metal foils using ultrasonic welding processes and evaluated their sensing characteristics. The authors reported that it was not possible to embed the bare fiber in copper. This was attributed to the high hardness of copper. The chemical plated fiber was embedded in aluminum; however, it was destroyed, because of the welding pressure, vibration and friction. The FBG fiber coated through a chemical-electroplating method was successfully embedded in aluminum, as presented in figure 6 [28], and showed preserved sensing character and function.

**Figure 7.**
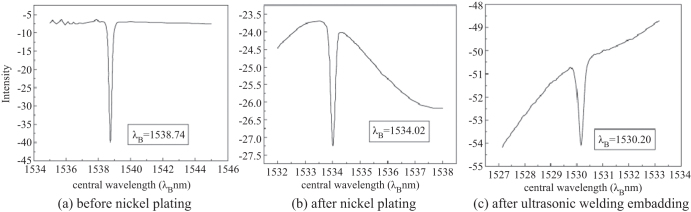
The Bragg peak of a FBG before nickel coating, after nickel plating, and after ultrasonic welding embedding. Reprinted from [28], copyright 2012, with permission from Elsevier.

The authors noted the preservation of the Bragg peak’s form after the coating and ultrasonic welding processes, as shown in figure 7. However, they reported that the Bragg peak wavelength was shifted down by about 4.72 nm after nickel chemical-electroplating, and further shifted down by 3.82 nm after ultrasonic welding embedding. They attributed this shift to the fact that the fiber is subjected to the axial compression due to the nickel deposition during the plating process and thermal contraction of the aluminum substrate during cooling. The authors recorded a difference in temperature sensitivity before and after embedding in the aluminum, which was attributed to the different thermal expansion coefficients of the aluminum (13 × 10^−6^ K^−1^ and 23 × 10^−6^ K^−1^) and the electroplated nickel layer (23 × 10^−6^ K^−1^). In addition, they experimentally confirmed that when tensile load (0–40 N) was applied on the embedded FBGs, they remained in good condition [28].

Rao *et al* [45] used an electroless plating method to coat FBG sensors. The observed common changes of the FBG spectrum after metalizing were reduced through a stress control technology, which enabled retention of the optical properties of the metal-coated FBGs. Zhang [44] developed a high thermal sensitivity optical FBG sensor with bi-material coating, which can be embedded into a smart tool. The author used laser-assisted maskless microfabrication and electroless plating to deposit a thin conductive silver layer on the FBG sensor followed by coating a thicker nickel layer on the silver layer. The coating has been shown to be effective for high temperature protection and thermal sensitivity enhancement.

## 3. Embedding processes

The novelty resides in the embedding techniques positioning the FBG sensors in optimal locations, allowing proper measurement coverage within the material. Cautions should be considered to maintain the integrity of the FBGs. The latter are capable of measuring pressure up to 100 MPa and temperature up to 300 °C without physical damage. The reflection spectrum of the FBG will experience a shift in the wavelength as a result of ultrasonic wave induced pressure. The FBGs can measure frequencies up to 40 MHz.

### 3.1. Ultrasonic consolidation

Embedding of optical fiber sensors in metallic materials is possible using ultrasonic consolidation (UC). The process is a novel manufacturing technique for fabrication of structures from metal foils. Being a solid-state process, it uses less ultrasonic energy and can be performed at low pressure and temperature compared to other technologies. Among the interesting applications of UC is the embedding of FBGs not only in plastic materials at lower energy (1.5–2 kW) but also within metal structures at 3–5 kW. The current limitation resides in the required smallest thickness (up to 2–3 mm) of the materials hosting the fiber. Kong and Soar [46] used the UC process for embedding optical fibers within aluminum structures in thin layers covering honeycomb structures. They reported that the optical fibers were fully embedded in the matrix material without damage.

**Figure 8.**
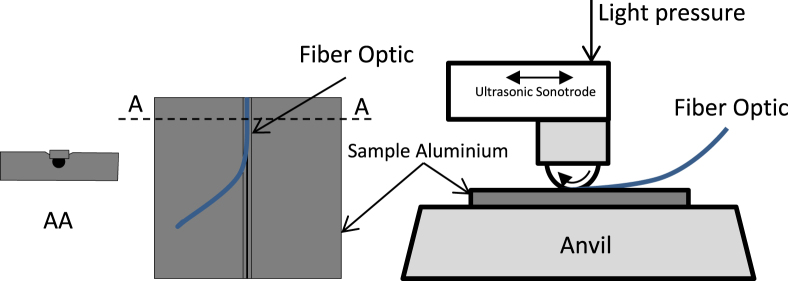
Proposed ultrasonic setup for embedding fiber optics in material.

**Figure 9.**
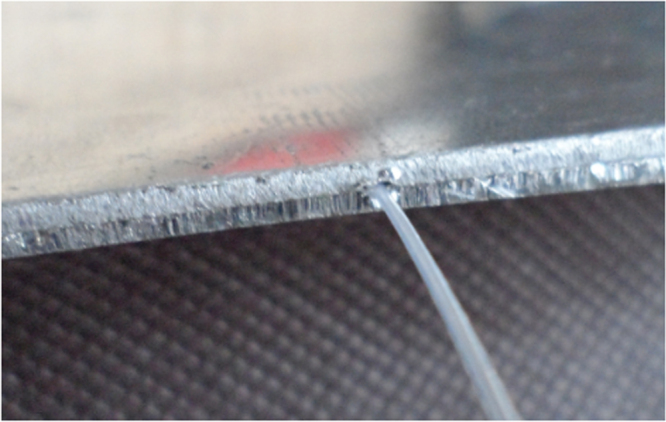
Embedded fiber between two aluminum plates.

We have carried out an initial trial (using the process principle shown in figure 8) by embedding a fiber between two aluminum plates of 0.5 mm thickness, as shown in figure 9, using an ultrasonic process at 20 kHz and 5 kW of power. There was a requirement to have a slit on one plate hosting the fiber with about 90 mm of length successfully embedded, then the fiber systematically breaks.

### 3.2. Laser-based layered manufacturing

The creation of complex parts and components is possible using layered manufacturing. Li *et al* [29] embedded FBG sensors into stainless steel structures using laser-assisted shape deposition manufacturing methodology. A thin conducting metallic layer was sputtered on the silica fiber. The fibers were electroplated with a Ni protective layer, and finally laser cladding with stainless steel was performed. The Ni/fiber interface showed full contact between the silica fibers and the Ni matrix of adequate thickness before and after laser cladding. The authors successfully performed strain and temperature measurements using the embedded FBG sensors. Alemohammad and Toyserkani [47] developed cutting tools with embedded sensors using laser-based layered manufacturing processes. The embedding process consisted of low temperature laser microdeposition of on-fiber silver thin films followed by nickel electroplating in a steel part, and finally a layer of tungsten carbide-cobalt was deposited through laser solid freeform fabrication. The authors [47] obtained a crack-free interface between the optical fiber and the silver layer as well as the silver and nickel layers. They found that the laser power has a prominent effect on the properties of the silver thin films and the mechanical properties. The embedded sensor, tested through a heating process on a hot plate, which simultaneously applied temperature and strain on the embedded FBG, showed that the sensor preserved its linear behavior implying a good integrity between the layers. The obtained temperature sensitivity was 25.8 pm °C^−1^ i.e. 2.58 times larger than that of a bare FBG (10 pm °C^−1^). This was attributed to the large coefficient of thermal expansion of the nickel metal part (13.4 × 10^−6^ °C^−1^) compared to steel (11.8 × 10^−6^ °C^−1^).

### 3.3. Innovative embedding processes

**Figure 10.**
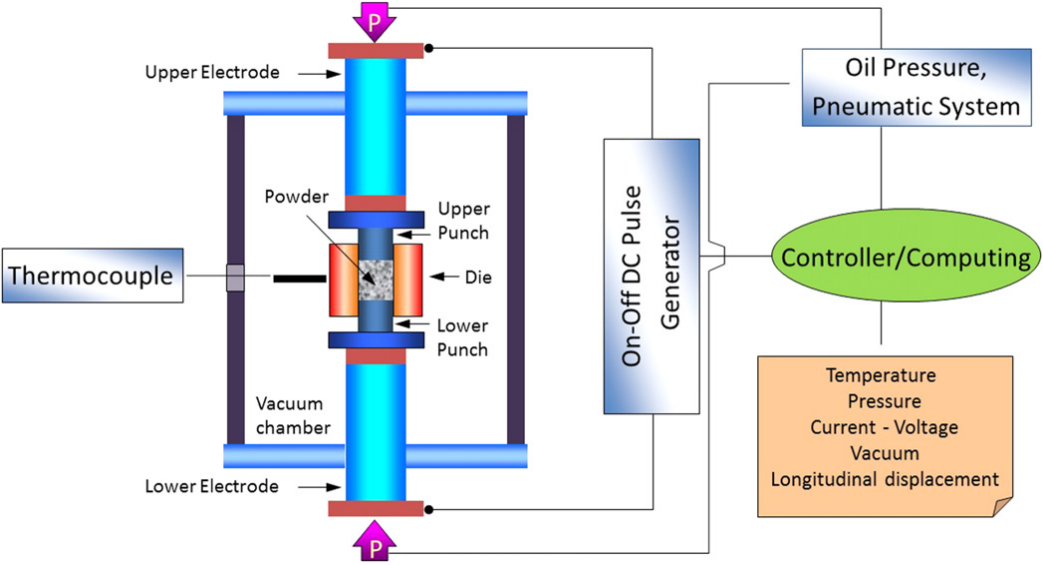
Schematic of a spark plasma sintering setup. Reproduced from [48].

Since FBG sensors cannot be embedded in metallic materials using high temperature processes, alternative novel low temperature processes may be used. Spark plasma sintering [48], as shown in figure 10, is one of the relatively low temperature processing techniques. It is a novel powder metallurgy method for processing fully dense metallic, ceramic, polymeric and composite materials. Furthermore, the process does not require a binder or pre-compaction step. The processing cycle involves application of low pressure and voltage, high intensity current, and is accomplished at low temperatures for a very short period compared to conventional processing methods.

**Figure 11.**
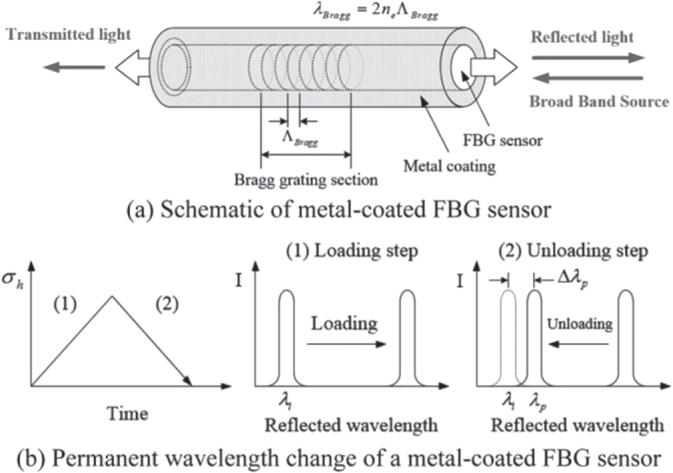
Principle of metal-coated FBG sensors. Reprinted from [49], copyright 2014, with permission from Elsevier.

However, because of spark discharge, a local high temperature-state may be generated momentarily. Therefore, the FBG sensor could be coated with a metallic layer, as shown in figure 11 [49], to protect it from damage as proved experimentally by Kim *et al* [49]. The authors concluded that despite the fact that coating may lead to adverse effects, the coated FBG sensors have a sufficient feasibility to be used as metal-coated optical fiber sensors.

### 3.4. Current challenges encountered in embedding sensors

The main challenges associated with the use of FBG sensors in composite materials for SHM were reviewed by Kinet *et al* [24]. The authors highlighted issues related to temperature–strain discrimination, demodulation of the amplitude spectrum, and connection between the embedded optical fibers and the surroundings. As for metals and alloys, the FBG sensor is highly delicate and would be damaged during high temperature embedding processes or by any small impact as it is brittle. Lengthy embedding is not systematic and needs investigation. Hence, embedding FBG sensors in metallic structures remains a challenging task. Furthermore, coating of FBG sensors with metallic films, which is a useful method to protect them from high temperature damage and improve their performance in harsh environments, also alters the sensor sensitivity [44]. In-depth embedding of sensors and actuators inside structural materials may trigger a concern for the integrity not only of the sensors and actuators but also of the material itself, as these will be considered as inclusions that may affect the overall performance. In this paper, considerations are about surface embedding rather than in-depth embedding. Surface embedded sensors have much less effect on the structural integrity.

## 4. Modeling and simulation

Modeling and simulation are important in any scientific research primarily for two reasons: (a) they reduce the cost of experimentation especially before a full-scale test, and (b) they allow one to study phenomena that are not possible to investigate using current experimental capabilities and techniques. An example of the first reason is the development of a large structure, e.g. an airplane, in which computational modeling is used throughout the design process for optimization and a full-scale test is performed on the complete structure at the end to ensure modeling predictions. On the other hand, a classical example of the second reason is understanding the effect of microstructure on the material’s macro behavior, e.g. the effect of dislocation structures, which is mainly studied using various simulation techniques as experimental capabilities do not allow the investigation of the microstructure at the required length scale. In the case of developing a nervous material, both reasons necessitate a modeling study as the nervous system will eventually be employed in structures and there is a need to understand the material’s response during and after manufacturing of such a nervous material, through possibly different routes. Depending on the manufacturing route and material used, there are certain issues that must be tackled using a modeling and simulation approach.

Using ultrasonic processing, one may embed sensors or sensor arrays on the surfaces of metals. The issues to be understood are the local plastic deformation occurring in the metal matrix, the flow of metal around the sensors, the nature of adhesive forces between the sensors and metal matrix, and the residual stresses around sensors. The plastic response of a metal is usually dependent on stress and strain along with the deformation temperature and strain rate, if applicable. In the case of ultrasonic welding, vibrational stress also affects the strain and strain rates. The other question that arises is related to the temperature reached in the ultrasonic weld zone and how it affects the joint and the sensors. Furthermore, it is understood that both friction and acoustic softening affect the ultrasonic processing of metals. The friction is classified as a surface effect and acoustic softening is classified as a volume effect, and both are not yet fully understood numerically or experimentally.

Very few studies [50–56] have been directed towards modeling ultrasonic machining processes. Gao and Doumanidis [50, 51] analyzed an ultrasonic bonding process for the case of spot welding for rapid prototyping applications. They were probably the first (considering also the MS thesis of Gao) to numerically model an ultrasonic welding process using the finite element method. They assumed a bilinear kinematic hardening material model with both linear elastic and plastic regions, each terminating at the yield strength and ultimate tensile strength respectively. Ding *et al* [52, 53] performed numerical analysis of wire bonding for electronics applications using ultrasonic processing. They investigated the effect of bonding parameters such as bond force and power on contact pressure, frictional energy and temperature rise. For comparison, they developed three-dimensional and two-dimensional finite element models. However, to reduce the computational time and cost, an equivalent 3D model is used in which the complete experimental setup is scaled down to reduce the size of the numerical problem. In addition, the focus is on understanding the surface effects and it was concluded that the frictional energy is one of the most important factors affecting the weld bond. A comparison of 2D and 3D models developed indicate that properly developed 2D models provide accurate results and can be used to reduce computational costs and time. It is also confirmed that the bulk temperature rise is not the main cause of ultrasonic wire bonding. Siddiq and Ghassamieh [54, 55] investigated the embedding of fibers in different aluminum alloys. Using the finite element method, they employed a thermo-mechanical cyclic plasticity model by Chaboche [56]. The total strain tensor *∊* is given as the sum of the elastic and plastic parts:

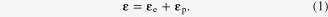


The stress tensor is expressed using an incremental form of Hooke’s law:

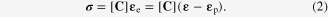


**Figure 12.**
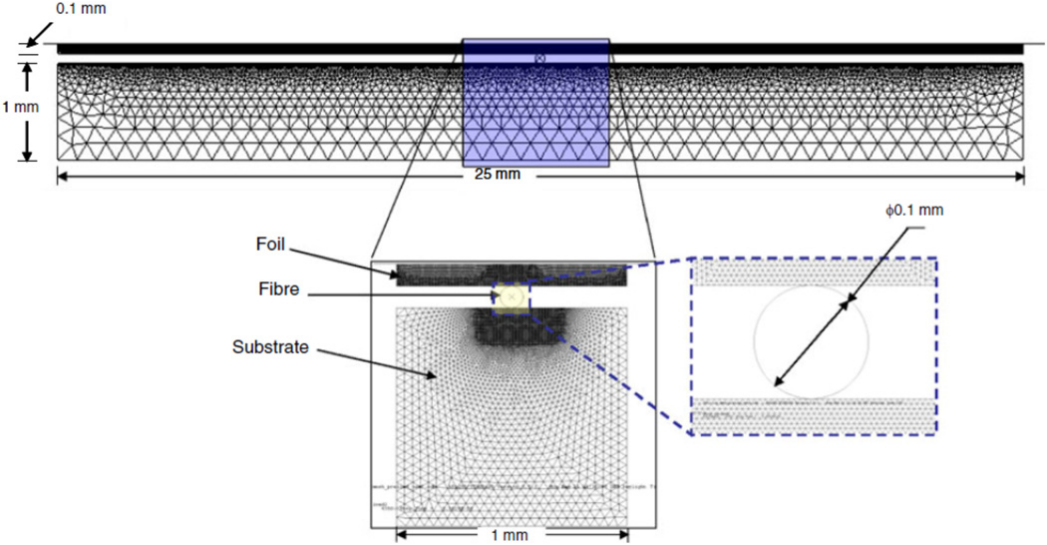
A typical problem setup for an ultrasonic consolidation model. Reproduced with permission from [54], copyright 2010, Springer–Verlag London Limited.

The flow rule to determine the plastic strain is given as:

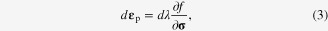
where *dλ* is the plastic multiplier and *f* is the yield criterion, defined as:

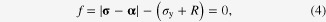
where ***α*** is the backstress due to kinematic hardening, *σ*_y_ is the initial yield stress and *R* is the isotropic hardening term given as:

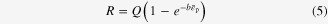
where 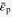 is the equivalent plastic strain, and *Q* and *b* are the material’s parameters. The evolution of backstress is predicted through:

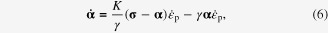
where *K* and *γ* are material parameters. The above model is modified to include thermal softening, introducing the following laws for isotropic and kinematic hardening:

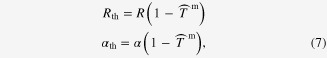
where 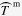 is the non-dimensional temperature defined in terms of melting temperature *T*_m_ and transition temperature *T*_t_ as: 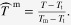 Furthermore, the above equations are modified to account for acoustic softening as:

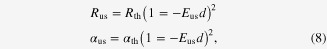
where *d* is the ultrasonic softening parameter. Siddiq and Ghassameiah [54, 55] started with a 2D model of size 25 mm × 1 mm with 0.1 mm diameter fiber to save computational cost and time. However, due to severe plastic deformation around the fiber, the mesh must be refined locally at the fiber surface. To achieve this with reasonable computational time, they used a smaller 2D model of size 1 mm × 1 mm with 0.1 mm diameter fiber. They specified model boundary conditions at the edges normal to the 25 mm length assuming that the region outside the 1 mm width does not affect the region considered for modeling. This problem setup is shown in figure 12 and is a different technique to the scaling down technique used by Kong *et al* [46] and Ding and Kim [53]. They also considered both the surface and volume effects. The conclusions indicate that a full embedding with metal flowing around the fiber is achieved. The temperature rise is much less than the melting point of the aluminum alloy considered and therefore the fiber will not be damaged during the embedding process. It is important to note that the friction work is the main source for ultrasonic welding without fiber embedding and in the case of embedding a fiber, the friction work reduces to 10 times lower than without fiber embedding.

**Figure 13.**
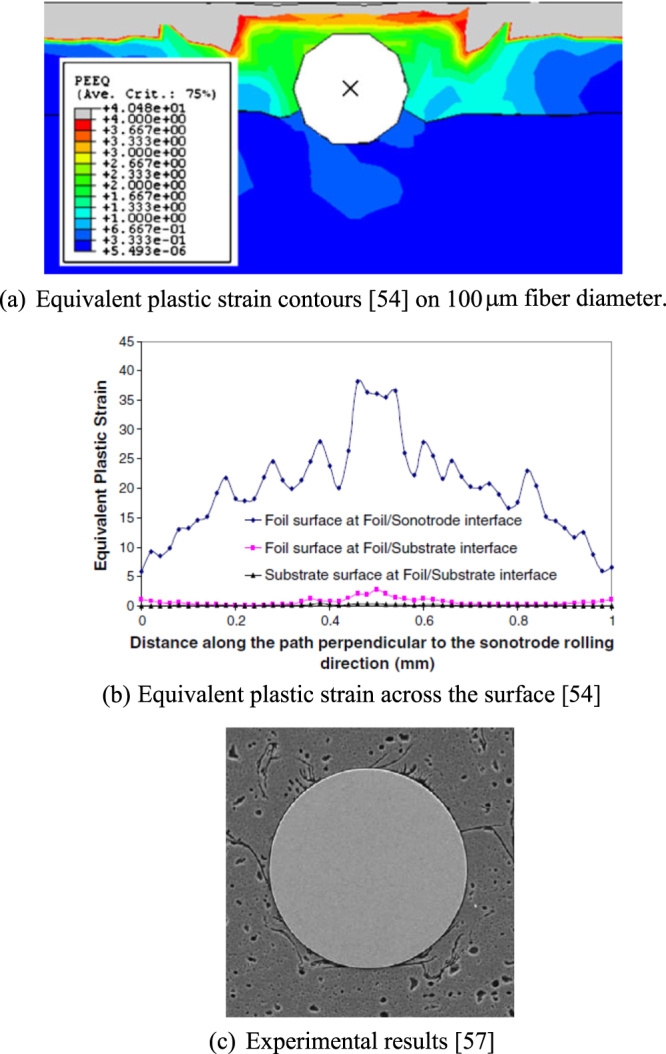
Numerical and experimental results. Parts (a) and (b) reproduced with permission from [54], copyright 2010, Springer-Verlag London Limited. Part (c) reprinted from [57], copyright 2005, with permission from Elsevier.

The temperature and friction work at the weld interface, consolidation of material around the fiber and the deformation during the ultrasonic consolidation process are important parameters to be investigated. Figure 13(a) shows contours of equivalent plastic strain and figure 13(b) shows its variation across the ultrasonically consolidated surface. As expected, higher levels of plastic strain are noted in the vicinity of the fiber and in the foil. The substrate shows the lowest levels of plastic deformation. Figure 13(c) shows experimental results of embedding a fiber in a metal substrate using ultrasonic consolidation. It must be noted, however, that the modeling results for embedding of shape memory alloy considers the fiber to be rigid. Significant deformation of optical fibers is possible and thus presents a technological challenge.

On the experimental side, shape memory alloy fibers, SiC fibers and optical fibers have been embedded in aluminum matrices [57–59] using an ultrasonic consolidation process. Major challenges include the state of plastic deformation of the parent material around the fibers and the degree of adhesion between the parent material and fibers. In addition, the integrity of the fiber being embedded is crucial. It is reported that SiC and shape memory alloy fibers can keep their integrity through the process parameters of ultrasonic consolidation, while optical fibers suffered deformation and partial to full loss of luminosity. It is also shown that out of the three welding parameters (oscillation amplitude, contact pressure, weld speed), oscillation amplitude carries the vital role in governing plastic deformation during the process. A relatively high amplitude along with low contact pressure excites the dislocations resulting in adequate plastic flow around the fibers. An amplitude of 8.5–12.5 *μ*m, a contact pressure of 205–275 kPa and a constant weld speed of 35 mm s^**−**1^ are deemed appropriate to achieve proper consolidation of aluminum foils and fibers [59].

The short process duration (4–9 ms) [60] hinders the study of ultrasonic treatment of materials. It is impossible to experimentally study the material’s behavior during this time. Therefore, another avenue could be the engagement of computational models, especially those based on a multi-scale approach, to investigate the changes occurring during the ultrasonic processing.

Two other fields of research on nervous materials are also important: analysis of the inclusions of various shapes in a matrix material and detecting/locating a force applied to a structure through the use of sensors’ readings. Several research works, which are not directed towards the development of nervous materials but could be used there, have been carried out on the investigation of deformation fields around inclusions. These include the numerical equivalent inclusion method [61], cubic inclusion arrangements [62], elliptical inclusion in finite composite laminate [63], the effects of non-metallic inclusions on fatigue strength of steels [64] and an elastic half-plane containing multiple inclusions [65]. The models on detection of an applied load (or stress) on a structure present an inverse problem, in which the displacement (usually) readings from sensors are used to precisely pinpoint the location of the applied load on the structure. Some representative publications include the study on reconstruction of a distributed force impacting an elastic plate [66], impact localization of reinforced plastic plates [67], impedance models for the monitoring of concrete using lead zirconate titanate (PZT) transducers [68, 69], determination of forces using fiber optic strain sensors [70] and the effect of sensor network placement on detection capability and accuracy [71].

The major issue in modeling the ultrasonic embedding process is the highly non-linear and multi-physics nature of the problem. Several models must be coupled together to successfully simulate the embedding process, which includes an acoustics model for the ultrasonic head, a thermo model for the local heat generated, interface modeling between the thin metal foil, optical fiber and the substrate metal, local temperature rise and the plastic deformation model. In addition, the size of the problem requires a very fine mesh especially around the optical fiber for a convergent numerical solution.

## 5. Concluding remarks and future directions

This review investigates a new class of materials with a key property of being nervous, i.e. emulating human feeling of surrounding environmental parameters. With multi-sensing and delaying failure capabilities, this innovative material will see tremendous applications in small- and large-scale designs.

The review has discussed several challenges encountered during this investigation. This includes suitable sensors, sensor embedding techniques, maintaining sensor integrity during embedding, the volumetric distribution of sensors to secure optimal coverage within the system under monitoring without compromising the material integrity, collection of data, and, power supply routes. Mechanisms of local self-healing within the material depending on the mode of failures, or inherent actuation techniques to at least delay any failure are of great importance. It goes without saying that cost will initially be a major factor.

Although most of the issues associated with FBG sensors in composite materials were solved, there are remaining issues still to be addressed, such as the connection aspect and curing cycle monitoring. The connection aspect is vital for the industrialization of the process; and this can be achieved through either free-space connection or designing a dedicated connector. Full control and monitoring of the curing cycle may bring about more benefits [19]. Promising results were reported on the possibility of embedding polymer optical fiber gratings [22] in composite materials at temperatures less than 70 °C [20, 21]. Research in this particular area should be focused on extending the use of polymer FBGs in high temperature environments.

The embedding process of FBG sensors within metallic structures for structural health monitoring is in its infancy; and it is believed to be a cutting-edge research topic. FBG sensor-embedded materials are critical in many applications including the machining tools, aerospace and automotive industries [46]. Maintaining the integrity of the optical sensors by protecting them from high temperature during the embedding process and achieving higher sensitivity of the optical sensor are the critical issues to be addressed [44].

The multi-physics nature of the ultrasonic embedding makes it highly non-linear, requiring the coupling of several models together to successfully simulate the phenomenon of embedding fibers using an ultrasonic technique. This process requires heavily computational models and therefore techniques must be used to reduce the computational effort without compromising the accuracy of the solution to study the stresses, strains and integrity of the nervous material. Furthermore, in order to find the location of the external stimuli on a nervous material, algorithms need to be developed for various geometries that can accurately deduce the location based on the readings from embedded sensors.

In summary, future research efforts should be centered on two important issues, i.e. embedding processes and protection of fibers while maintaining their sensing capability. In addition to exploring the possibility of introducing innovative processing techniques such as spark plasma sintering, there is a strong need to optimize the processing parameters of existing methods such as ultrasonic consolidation and laser-based layered manufacturing. The possibility of extending the use of polymer optical fiber gratings in harsh environments should be explored. Self-healing mechanisms could be embedded together in proximity to the sensors to correct for any possible failures, and would delay failures for a limited period, to avoid an unexpected instant accident. This will have a huge impact on equipment and public safety.
